# Decreased brain functional connectivity associated with cognitive dysfunction in women with second pregnancy

**DOI:** 10.3389/fnagi.2022.963943

**Published:** 2022-07-22

**Authors:** Juan Zhang, Tao Zhang, Yu-Chen Chen, Huiyou Chen, Yuan Feng, Wen-Wei Tang, Jin-Xia Zheng

**Affiliations:** ^1^Department of Neurology, Nanjing Yuhua Hospital, Yuhua Branch of Nanjing First Hospital, Nanjing, China; ^2^Department of Radiology, Nanjing Maternity and Child Health Care Hospital, Women’s Hospital of Nanjing Medical University, Nanjing, China; ^3^Department of Radiology, Nanjing First Hospital, Nanjing Medical University, Nanjing, China

**Keywords:** parous women, primiparous women, posterior cingulate cortex, resting-state fMRI, cognitive dysfunction

## Abstract

**Purpose:**

Previous research has found that women with second pregnancy may have an increased risk of cognitive dysfunction. This study aims to investigate the intrinsic functional connectivity (FC) pattern of the DMN anchored on posterior cingulate cortex (PCC) in postpartum women, especially the parous women using resting-state functional magnetic resonance imaging (rs-fMRI).

**Methods:**

Twenty parous women, 26 primiparous women, and 30 nulliparous women were included for rs-fMRI scan. They were age and education well matched. A seed based FC method was conducted to reveal FC patterns with other brain regions using a region of interest in the PCC. The relationships between FC patterns and cognitive performance were further detected.

**Results:**

Relative to primiparous women, parous women had significantly decreased FC primarily between the PCC and the right middle frontal gyrus and right parahippocampal gyrus. The decreased FC to the right parahippocampal gyrus in parous women was positively associated with the reduced DST scores (rho = 0.524, *p* = 0.031). Moreover, parous women compared with nulliparous women showed significantly decreased FC between the PCC and the left superior frontal gyrus and left middle frontal gyrus. The reduced FC to the left superior frontal gyrus in parous women was also positively associated with the lower DST scores (rho = 0.550, *p* = 0.022).

**Conclusion:**

Our result highlights that women with second pregnancy revealed decreased FC between the DMN regions with the parahippocampal gyrus and prefrontal cortex, which was correlated with specific impaired cognitive function. This study may provide new insights into the neuropathological mechanisms of postpartum cognitive impairment and enhance our understanding of the neurobiological aspects during postpartum period.

## Introduction

The universal two-child policy has been conducted in China for a long time since October, 2015 (Zeng and Hesketh, 2016). Women with second pregnancy may face multiple complex psychological challenges owing to the abrupt policy changes (Priya et al., 2018). In addition to postpartum depression, anxiety or other mental disorders, women may show an increased risk of cognitive dysfunction, which primarily manifests as poor memory, forgetfulness, difficulty concentrating, and distractibility (Christensen et al., 2010; Postma et al., 2014; Albin-Brooks et al., 2017). Prior studies have found that postpartum women show cognitive impairment that may occur prior to affective disorder (Christensen et al., 2010; Postma et al., 2014; Meena et al., 2016). However, the etiology of the neurophysiological mechanism of this increased risk remains still unclear.

Multimodal neuroimaging techniques have been used to investigate the brain structural and functional alterations in primiparous women, which are linked with cognitive dysfunction (Kim et al., 2010; Postma et al., 2014; Hoekzema et al., 2017; Zheng et al., 2018, 2020). Hoekzema et al. (2017) confirmed that primiparous women exhibited a symmetrical pattern of extensive gray matter (GM) reductions throughout pregnancy, mainly affecting the bilateral lateral prefrontal and temporal cortex. Nonetheless, no study is reported regarding the brain network alterations in the central nervous system of women with second pregnancy.

Using resting-state functional magnetic resonance imaging (rs-fMRI), low frequency fluctuations (<0.01 Hz) in blood oxygenation level-dependent (BOLD) signal during rest reflect spontaneous neural activity can be conceptualized as a network of anatomically linked regions (Mantini et al., 2007; Lee et al., 2013). Previous studies have used rs-fMRI to examine the abnormal brain activity and network in postpartum depression (Xiao-Juan et al., 2011; Chase et al., 2013; Deligiannidis et al., 2013; Fisher et al., 2016; Zhang et al., 2020). Furthermore, the default mode network (DMN) shows consistently higher BOLD activity during rest in several brain regions, such as the posterior cingulate cortex (PCC), medial prefrontal cortex (mPFC), and anterior cingulate cortex (ACC) (Greicius et al., 2003). The DMN acts a pivotal role in various cognitive functions, including memory, prospection, and self-processing (Spreng and Grady, 2010). Among these regions, the PCC is a key region with strong connectivity with the hippocampal memory system, which is involved in autobiographical memory, and imagining the future as well as spatial navigation (Leech and Sharp, 2014). During cognitive processing, the PCC is functionally linked to the DMN regions, such as the mPFC (Fransson and Marrelec, 2008; Li et al., 2020). Several studies have reported reduced resting-state functional connectivity (FC) in DMN regions involved in social cognition in postpartum women (Xiao-Juan et al., 2011; Chase et al., 2013; Zheng et al., 2020). Moreover, aberrant brain activity of the PCC in postpartum women has also been detected in prior studies (Zheng et al., 2018; Bak and Nah, 2021). However, how DMN dysfunction draws a relation to cognitive symptoms in parous women compared with primiparous women still remains unknown.

To address this problem, this study attempted to explore and analyze the resting-state FC analysis of the PCC in the brain between postpartum women and nulliparous women. We determine whether parous women exhibited aberrant FC pattern of the DMN using a seed-based approach. Moreover, the relationships between abnormal DMN connectivity and cognitive dysfunction were further investigated in parous women compared with primiparous women. We assumed that the parous women might show disrupted brain FC that was associated with cognitive dysfunction compared with primiparous women or nulliparous women. Our findings might provide new insights into neurophysiological mechanisms of cognitive impairment in women with second pregnancy.

## Materials and methods

### Participants

A total of 76 women (aged between 20 and 40 years, right-handed, ≥9 years of education) made up of 20 parous women, 26 primiparous women, and 30 nulliparous women were included through community health screening, which were age and education-matched. No subject was subsequently excluded because of the exceeded limits for head motion during scanning. All the parous and primiparous women were medication free and had delivered a healthy and full-term infant in the preceding 3 months. None of the women experienced any complications during pregnancy or delivery, such as hypertension, heart disease, eclampsia, diabetes, or postpartum hemorrhage. This study was approved by the Research Ethics Committee of the Nanjing Medical University. All the subjects provided written informed consent before participation.

Subjects were excluded if they suffered from severe smoking, alcoholism, Alzheimer’s disease, Parkinson’s disease, stroke, brain trauma, major depression, epilepsy, and MRI contraindications. None of the women had postnatal depression according to the Edinburgh Postnatal Depression Scale (EPDS, overall scores <12) (Cox et al., 1987).

Blood samples were collected by venepuncture at 8 A.M. to assess the levels of fasting plasma glucose (FPG), triglycerides, total cholesterol, low density lipoprotein (LDL)-cholesterol, and high-density lipoprotein (HDL)-cholesterol, in order to exclude the hyperglycemia and hyperlipidemia at the time of examination.

The neuropsychological status of the participants was established using the Mini Mental State Exam (MMSE), Montreal Cognitive Assessment (MoCA), Complex Figure Test (CFT), Clock-Drawing Test (CDT), Verbal Fluency Test (VFT), Digit Span Test (DST), Auditory Verbal Learning Test (AVLT), Trail-Making Test (TMT) A and B, Digit Symbol Substitution Test (DSST), Self-Rating Depression Scale (SDS), and Self-Rating Anxiety Scale (SAS).

### Magnetic resonance imaging acquisition

Magnetic resonance imaging data were acquired using a 3.0 Tesla MRI scanner (Ingenia, Philips Medical Systems, Netherlands) with an 8-channel receiver array head coil. During scanning, the subjects were supposed to lie quietly with their eyes closed and avoid head movement, but not to fall asleep or think about anything special. Structural images were obtained using a three-dimensional turbo fast echo (3D-TFE) T1WI sequence as follows: repetition time (TR) = 8.1 ms; echo time (TE) = 3.7 ms; slices = 170; thickness = 1 mm; gap = 0 mm; flip angle (FA) = 8^°^; acquisition matrix = 256 × 256; field of view (FOV) = 256 mm × 256 mm. Fluid-attenuated inversion recovery (FLAIR) were also acquired: TR = 7000 ms; TE = 120 ms; slices = 18; thickness = 6 mm; gap = 1.3 mm; FA = 110^°^; and voxel size = 0.65 mm × 0.95 mm × 6 mm. Functional images were acquired using a gradient echo-planar imaging (EPI) sequence as follows: TR = 2000 ms; TE = 30 ms; slices = 36; thickness = 4 mm; gap = 0 mm; FOV = 240 mm × 240 mm; acquisition matrix = 64 × 64; and FA = 90^°^.

### Functional data analysis

Functional magnetic resonance imaging data preprocessing was performed using Data Processing and Analysis for (Resting-State) Brain Imaging (DPABI_V6.1_220101)^1^ with the following procedures. Removed the first ten volumes function images, slice timing effects and head motion corrected. The remaining dataset was spatially normalized to the Montreal Neurological Institute (MNI) 152 space (resampling voxel size = 3 mm × 3 mm × 3 mm). Moreover, smoothing with an 8-mm full width at half-maximum Gaussian kernel, detrending and filtering (0.01–0.08 Hz) were performed.

The seed regions of interest (ROI) of the PCC were generated from Brodmann template using the WFU_PickAtlas software (Maldjian et al., 2003). For seed based FC analysis, correlation analysis of time course was performed between the spherical seed region (PCC) and each voxel of the whole brain for each subject using DPABI software. Six head motion parameters and mean time courses of global, WM and CSF signals were included in the regression analysis.

### Structural analysis

Structural images were processed using the VBM toolbox in SPM12.^2^ Briefly, the structural images were normalized and segmented into GM, white matter (WM), and cerebrospinal fluid (CSF) in SPM12. The GM, WM, and brain parenchyma volume were divided by the total intracranial volumes. T1 images were normalized to the MNI template using affine linear registration followed by Gaussian smoothing (FWHM = 8 mm).

### Statistical analysis

Differences in demographic information and clinical measures were analyzed using one-way analysis of variance (ANOVA) among the three groups followed by a *post-hoc* test (*t*-test for means and χ^2^-test for proportions). The SPSS software (version 25.0, Chicago, IL, United States) was used for statistical analyses and *p* < 0.05 was considered significant.

To investigate the between-group FC differences, *post-hoc* analysis was further conducted by one-way ANOVA. Significant thresholds were corrected using false discovery rate (FDR) criterion and set at *p* < 0.01. Age, education, and GM volume were used as nuisance covariates to adjust for the effect of these factors. To identify the relationship between aberrant FC and cognitive variables, the mean *z*-values of each brain region that showed significant group differences were calculated within every subject. Spearman correlation analysis between the mean *z*-values and each variable were performed using SPSS software. The statistical threshold was set at *p* < 0.05. Partial correlations were performed after correction for age, education, and GM volume. Bonferroni correction was applied for multiple comparisons in the correlation analyses.

## Results

### Clinical data analysis

The demographic characteristics and neuropsychological results of the parous women, primiparous women and nulliparous women were shown in Table 1. The three groups did not significantly differ in terms of age, education, FPG, blood lipids and WM hyperintensity (all *p* > 0.05). The parous women had significantly poorer DST scores than the primiparous women (*p* < 0.05). The other neuropsychological tests exhibited no significant differences among parous women, primiparous women and nulliparous women.

**TABLE 1:** Demographics and neurocognitive characteristics of the parous, primiparous, and nulliparous women.

	Parous women (*n* = 20)	Primiparous women (*n* = 26)	Nulliparous women (*n* = 30)	*p*-value
Age (year)	32.15 ± 3.13	30.15 ± 2.62	30.53 ± 3.88	0.111^a^
Education levels (years)	17.55 ± 2.09	17.15 ± 1.74	16.77 ± 3.15	0.546^a^
Fasting glucose (mmol/L)	4.73 ± 0.32	4.84 ± 0.45	4.87 ± 0.38	0.428^a^
Triglycerides (mmol/L)	0.70 ± 0.26	0.83 ± 0.32	0.73 ± 0.26	0.275^a^
Total cholesterin (mmol/L)	4.23 ± 0.54	4.42 ± 0.73	4.18 ± 0.57	0.328^a^
LDL-cholesterin (mmol/L)	2.43 ± 0.42	2.60 ± 0.63	2.36 ± 0.44	0.057^a^
HDL-cholesterin (mmol/L)	1.28 ± 0.17	1.47 ± 0.34	1.38 ± 0.24	0.244^a^
WM hyperintensity	0 (0–1)	0 (0–2)	0 (0–2)	0.578^a^
**Cognitive performance**				
MMSE	28.85 ± 1.27	29.00 ± 0.94	29.17 ± 0.79	0.522^a^
MoCA	25.85 ± 1.81	26.23 ± 1.18	26.30 ± 1.09	0.484^a^
AVLT	31.11 ± 8.12	34.62 ± 8.34	35.50 ± 7.68	0.194^a^
AVLT-delayed recall	6.30 ± 2.36	7.19 ± 2.15	6.97 ± 2.24	0.394^a^
CFT	34.43 ± 1.70	34.58 ± 1.67	34.48 ± 1.78	0.955^a^
CFT-delayed recall	17.05 ± 2.96	16.73 ± 2.95	17.60 ± 2.01	0.458^a^
DST	11.45 ± 1.47	13.73 ± 2.96	14.43 ± 3.31	0.002*^a^
TMT-A	53.75 ± 17.45	50.12 ± 17.26	48.30 ± 13.28	0.493^a^
TMT-B	100.45 ± 39.81	86.96 ± 14.67	103.63 ± 34.10	0.098^a^
CDT	3.60 ± 0.60	3.42 ± 0.50	3.57 ± 0.50	0.465^a^
VFT	14.40 ± 4.11	15.81 ± 3.24	13.67 ± 3.75	0.099^a^
DSST	67.55 ± 8.10	69.00 ± 9.57	69.17 ± 8.24	0.792^a^
EPDS	4.85 ± 1.66	4.18 ± 2.52	–	0.322^b^
SDS	38.55 ± 5.74	38.88 ± 6.40	38.50 ± 5.62	0.968^a^
SAS	37.35 ± 6.00	40.00 ± 7.56	39.40 ± 7.24	0.430^a^

Data are represented as Mean ± SD, n (%) or median (range), *p < 0.05. For comparisons of demographics: ^a^P-values were obtained by using one-way ANOVA tests; ^b^P-values were obtained using two-sample t-test. LDL, low-density lipoprotein; HDL, high-density lipoprotein; WM, white matter; FD, framewise displacement; MMSE, mini mental state exam; MoCA, Montreal cognitive assessment; AVLT, auditory verbal learning test; CFT, complex figure test; DST, digit span test. TMT-A, trail making test-part A; TMT-B, trail making test-part B; CDT, clock drawing test; VFT, verbal fluency test; DSST, digit symbol substitution test; EPDS, Edinburgh postnatal depression scale; SDS, self-rating depression scale; SAS, self-rating anxiety scale; WMH, white matter hyperintensity.

### Structural data analysis

Table 2 presents the comparisons of the whole-brain volumes (GM volume, WM volume, and brain parenchyma volume) among parous women, primiparous women and nulliparous women. The results showed that there were no significant changes in GM and WM volumes among the three groups (*p* > 0.05).

**TABLE 2:** Structural results among the parous, primiparous, and nulliparous women.

	Parous women (*n* = 20)	Primiparous women (*n* = 26)	Nulliparous women (*n* = 30)	*p*-value
Gray matter volume (% of TIV)	32.0 ± 1.6	31.6 ± 1.7	32.2 ± 1.3	0.294
White matter volume (% of TIV)	29.8 ± 1.5	29.4 ± 1.5	29.5 ± 1.5	0.731
Brain parenchyma volume (% of TIV)	61.8 ± 2.7	61.0 ± 2.8	61.7 ± 2.3	0.510

Data are expressed as mean ± SD. TIV, total intracranial volume.

### Functional data analysis

#### Parous women vs. primiparous women

**FIGURE 1:**
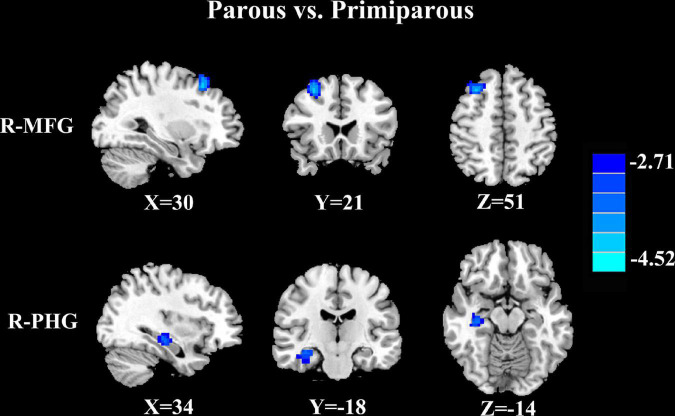
Compared with primiparous women, parous women showed significantly decreased FC between the PCC and the right middle frontal gyrus (R-MFG) and right parahippocampal gyrus (R-PHG) (*p* < 0.01, FDR corrected).

Compared with primiparous women, parous women showed significantly decreased FC between the PCC and the right middle frontal gyrus and right parahippocampal gyrus (Figure 1 and Table 3, *p* < 0.01, FDR corrected).

**TABLE 3:** Decreased FC of PCC in parous women compared to primiparous women.

Brain region	BA	MNI coordinates x, y, z (mm)	T score	Voxels
Right middle frontal gyrus	8	30, 21, 51	−4.4567	93
Right parahippocampal gyrus	36	34, −18, −14	−3.0508	120

Thresholds were set at a corrected p < 0.01 corrected by FDR criterion. BA, Brodmann’s area; MNI, Montreal neurological institute.

#### Parous women vs. nulliparous women

**FIGURE 2:**
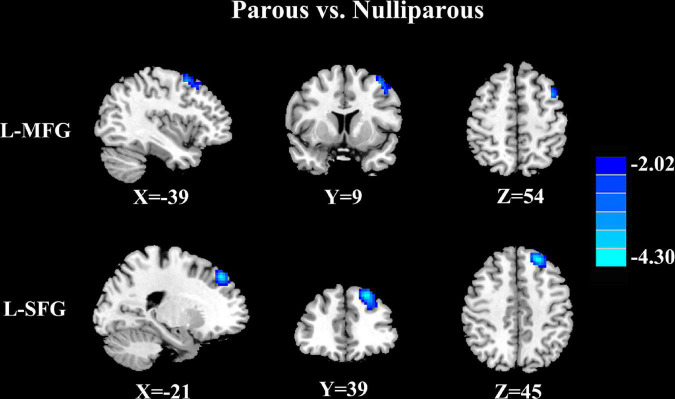
Parous women compared with nulliparous women exhibited significantly reduced FC between the PCC and the left superior frontal gyrus (L-SFG) and left middle frontal gyrus (L-MFG) (*p* < 0.01, FDR corrected).

Parous women compared with nulliparous women exhibited significantly reduced FC between the PCC and the left superior frontal gyrus and left middle frontal gyrus (Figure 2 and Table 4, *p* < 0.01, FDR corrected).

**TABLE 4:** Decreased FC of PCC in parous women compared to nulliparous women.

Brain region	BA	MNI coordinates x, y, z (mm)	T score	Voxels
Left middle frontal gyrus	6	−39, 9, 54	−3.4509	69
Left superior frontal gyrus	8	−21, 39, 45	−4.2416	218

Thresholds were set at a corrected p < 0.01 corrected by FDR criterion. BA, Brodmann’s area; MNI, Montreal neurological institute.

#### Primiparous women vs. nulliparous women

**FIGURE 3:**
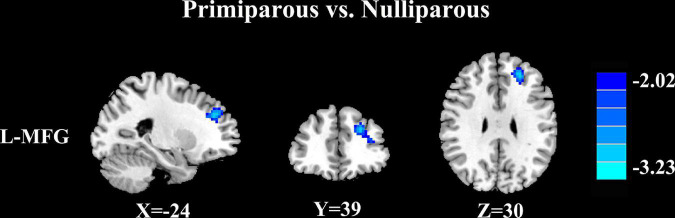
Relative to nulliparous women, primiparous women revealed a significant reduction in FC between the left middle frontal gyrus (L-MFG) (*p* < 0.01, FDR corrected).

Relative to nulliparous women, primiparous women revealed a significant reduction in FC between the left middle frontal gyrus (Figure 3 and Table 5, *p* < 0.01, FDR corrected).

**TABLE 5:** Decreased FC of PCC in primiparous women compared to nulliparous women.

Brain region	BA	MNI coordinates x, y, z (mm)	T score	Voxels
Left middle frontal gyrus	10	−24, 39, 30	−3.2027	137

Thresholds were set at a corrected p < 0.01 corrected by FDR criterion. BA, Brodmann’s area; MNI, Montreal neurological institute.

### Correlation results

**FIGURE 4:**
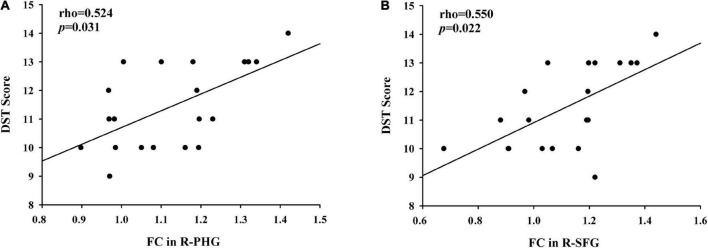
**(A)** Compared with primiparous women, the decreased FC of the PCC to the right parahippocampal gyrus in parous women was positively associated with the reduced DST scores (rho = 0.524, *p* = 0.031); **(B)** compared with nulliparous women, the decreased FC of the PCC to the left superior frontal gyrus in parous women was positively associated with the lower DST scores (rho = 0.550, *p* = 0.022).

Compared with primiparous women, the decreased FC of the PCC to the right parahippocampal gyrus in parous women was positively associated with the reduced DST scores (rho = 0.524, *p* = 0.031) (Figure 4A). Moreover, compared with nulliparous women, the decreased FC of the PCC to the left superior frontal gyrus in parous women was also positively associated with the lower DST scores (rho = 0.550, *p* = 0.022) (Figure 4B). These correlations were corrected for age, education, and GM volume. None of the other decreased FC was correlated with other cognitive performances.

## Discussion

As the central region of the DMN, the PCC performs diverse cognitive functions including visuospatial memory and processing of emotional and non-emotional information (Vogt et al., 2006; Leech and Sharp, 2013; Sestieri et al., 2017). Moreover, the DST score was used to assess the immediate memory (Hale et al., 2002). Therefore, the correlation between decreased PCC activity and impaired DST scores may indicate the decline of the short-term memory in postpartum women. The PCC is also recognized for its role in self-referential processing and social cognition (Mars et al., 2012). Alterations in endogenous sex steroid hormone levels during the postpartum period may result in widespread neural changes, including in the PCC (Fisher et al., 2016). Furthermore, our previous study had mainly demonstrated decreased resting-state FC patterns within the DMN regions, especially the PCC, which were associated with impaired cognitive function in primiparous women without depression (Zheng et al., 2020). Therefore, our findings indicate that reduced PCC connectivity pattern within the DMN may be responsible for the impaired short-term memory in parous women, which is different from that in primiparous women.

Furthermore, reduced FC of the PCC to the right parahippocampal gyrus in parous women was positively associated with the impairment DST scores. The parahippocampal area has been hypothesized to play a critical role in memory recollection and transferring information from the hippocampus to the association areas (Diederen et al., 2010). Women with postpartum psychosis showed smaller gray matter volume and decreased surface area in parahippocampal gyrus compared to women without postpartum psychosis (Fusté et al., 2017). Sharma et al. (2021) demonstrated disrupted FC between the posterior DMN and parahippocampal gyrus in older adults with subjective cognitive decline and correlates with subjective memory ability. Our results suggested that the FC of the PCC to the parahippocampal gyrus is another connection route associated with short-term memory. Since PCC–parahippocampal cortical connections provide access to autobiographical and other memories involved in self/other-relevant thought, women with second pregnancy may be less apt to access these cognitive functions.

The prefrontal cortex, especially the superior and middle frontal cortex, is mainly responsible for executive function (Yuan and Raz, 2014). We found decreased FC between PCC and prefrontal cortex were linked to impaired DST performance in parous women, which may indicate the dysfunction of executive abilities. Prior studies have detected disrupted executive function as one of the main cognitive dysfunction in postpartum women (Anderson and Rutherford, 2012; Meena et al., 2016). A fMRI study showed that the prefrontal brain activity during a response inhibition task was decreased throughout the first postpartum weeks in healthy women (Bannbers et al., 2013). Our combined ALFF and ReHo analyses revealed reduced neuronal activity, mainly in the PCC and prefrontal cortex, which was associated with executive dysfunction in primiparous women (Zheng et al., 2018). Furthermore, compared with the nulliparous women, postpartum women had a significantly decreased FC between the PCC and the left mPFC (Zheng et al., 2020). The current study extends the work that aberrant brain connectivity in the prefrontal cortex may play a pivotal role in postpartum cognitive impairment, especially the executive dysfunction.

This study has several limitations. First, the relatively limited sample size may lead to statistical bias and may reduce the ability to detect a relationship between abnormal FC and cognitive decline in parous women. Further studies with larger sample sizes are required in the future. Second, we only selected the PCC as the ROI but did not analyze FC changes in the other regions of the DMN. Therefore, future work should further analyze FC changes within the DMN in postpartum women. Third, the low temporal resolution and hemodynamic response delay of fMRI may affect the connectivity results. Studies on the combination of brain structure at the molecular or cellular level will be helpful to further explore the underlying neural mechanisms of cognitive dysfunction after pregnancy. Finally, physiologic noise, including respiratory, head motion, and cardiac fluctuations, might have compromised our results. These confounding factors should be taken into account in future studies.

## Conclusion

Taken together, this study showed that women with second pregnancy may cause FC alterations between the DMN regions with the parahippocampal gyrus and prefrontal cortex. Significant associations were also found between the disrupted FC networks and cognitive dysfunction in parous women. These results may provide new insights into the neuropathological mechanisms of cognitive impairment during postpartum period, especially for the parous women. The second pregnancy is associated with potential brain connectivity network alterations, which may provide more pivotal information for the clinical obstetrics about the early prevention from postpartum cognitive dysfunction.

## Data availability statement

The original contributions presented in this study are included in the article/supplementary material, further inquiries can be directed to the corresponding authors.

## Ethics statement

The studies involving human participants were reviewed and approved by the Research Ethics Committee of the Nanjing Medical University. The patients/participants provided their written informed consent to participate in this study.

## Author contributions

JZ and TZ designed the experiment, collected the data, performed the analysis, and wrote the manuscript. Y-CC, HC, and YF collected the data. W-WT and J-XZ contributed to the discussion and manuscript revision. All authors contributed to the article and approved the submitted version.

## Conflict of interest

The authors declare that the research was conducted in the absence of any commercial or financial relationships that could be construed as a potential conflict of interest.

## Publisher’s note

All claims expressed in this article are solely those of the authors and do not necessarily represent those of their affiliated organizations, or those of the publisher, the editors and the reviewers. Any product that may be evaluated in this article, or claim that may be made by its manufacturer, is not guaranteed or endorsed by the publisher.

## Footnotes

1
http://rfmri.org/dpabi

2
http://www.fil.ion.ucl.ac.uk/spm
